# Nanoparticle-Mediated Therapy with miR-198 Sensitizes Pancreatic Cancer to Gemcitabine Treatment through Downregulation of VCP-Mediated Autophagy

**DOI:** 10.3390/pharmaceutics15082038

**Published:** 2023-07-28

**Authors:** Christian Marin-Muller, Dali Li, Jian-Ming Lü, Zhengdong Liang, Osvaldo Vega-Martínez, Sue E. Crawford, Mary K. Estes, William E. Fisher, Changyi Chen, Qizhi Yao

**Affiliations:** 1 Michael E. DeBakey Department of Surgery, Baylor College of Medicine, Houston, TX 77030, USA; christian@speratum.com (C.M.-M.);; 2 Department of Molecular Virology and Microbiology, Baylor College of Medicine, Houston, TX 77030, USA; 3 Speratum Biopharma, Inc., Dover, DE 19901, USA; 4 Center for Translational Research on Inflammatory Diseases (CTRID), Michael E. DeBakey VA Medical Center, Houston, TX 77030, USA

**Keywords:** microRNA-198, pancreatic cancer, VCP, gemcitabine, drug resistance, nanoparticles, autophagy, RNAi therapeutic

## Abstract

Pancreatic ductal adenocarcinoma (PDAC) remains an extremely aggressive disease characterized by rapidly acquired multi-drug resistance, including to first-line chemotherapeutic agent gemcitabine. Autophagy is a process that is often exploited by cancer and is one of several intrinsic factors associated with resistance to gemcitabine. We have previously found that miR-198 acts as a tumor suppressor in PDAC through the targeting of factors including Valosin-containing protein (VCP). VCP has been reported to play an important role in autophagic flux. In this study, we investigated whether the repression of VCP through miR-198 administration disrupts the autophagy process and sensitizes PDAC cells to gemcitabine treatment in vitro. Moreover, we used LGA-PEI (LPNP) nanoparticles to effectively administer miR-198 to tumors in vivo, inducing tumor sensitization to gemcitabine and leading to a significant reduction in tumor burden and metastases and a concomitant downregulation of VCP expression and autophagy maturation. Our results indicate a potential therapeutic strategy for targeting gemcitabine resistant PDAC and establishes the use of LPNPs for effective therapeutic delivery of nucleic acids in vitro and in vivo.

## 1. Introduction

Pancreatic ductal adenocarcinoma (PDAC) remains an extremely aggressive disease for which no effective chemotherapeutic modality has been found. While several chemotherapy regimens have been approved for metastatic PDAC, the most widely used agent is gemcitabine—a nucleoside analogue first approved by the FDA for metastatic PDAC in 1996 [1]. While gemcitabine treatment can prolong the median survival time and provide benefits to patients, it presents only a 5.4% partial response rate with a progression-free survival interval of less than 4.2 months [2], a result of innate or acquired multi-drug resistance characteristic of pancreatic tumors [3]. The mechanism of PDAC cell resistance to chemotherapeutic agents is not clearly understood, but several intrinsic factors have been found to be associated with gemcitabine resistance in PDAC [4]. These factors include alterations in molecules associated with gemcitabine transport and metabolism, such as the nucleoside transporter-1 (hENT1) [5], activation of tyrosine kinase genes such as c-Src [6], and aberrant expression of cellular survival genes, such as those involved in the phosphatidylinositol 3-kinase/Akt (PI3K/Akt) pathway [7] and alterations associated with the tumor microenvironment, among others [8,9]. Several studies have also identified an association between gemcitabine resistance and increased autophagy in PDAC cells [10,11,12]. Pancreatic cancer cells exhibit constitutive autophagy under basal conditions, being required for malignant transformation in KRAS-driven tumors [13]. Autophagy is additionally induced in PDAC cells after gemcitabine treatment, highlighting the importance of autophagic flux in acquiring resistance to gemcitabine in pancreatic cancer tumor cells [10,14]. Furthermore, genetic, or pharmacological disruption of the autophagy process have been found to enhance the sensitivity of drug- and radiation-induced cytotoxicity in preclinical studies in various types of cancer, both in vitro and in vivo [15,16,17]. In pancreatic cancer, autophagy disruption has been found to enhance the cytotoxic effects of gemcitabine [10,11]. These results have led to several clinical trials, which have had variable responses for reasons that are not abundantly clear [18]. Most recently, a randomized Phase II study demonstrated that the addition of hydroxychloroquine to neoadjuvant gemcitabine and nab-paclitaxel chemotherapy in patients with resectable PDAC resulted in inhibition of autophagy and resulting improved response, indicating the importance of autophagy in PDAC chemotherapeutic resistance [19]. These and other recent encouraging results have renewed interest in identifying novel potential inhibitors of PDAC-mediated autophagy.

MicroRNAs (miRNAs) are small non-coding RNAs of approximately 19–25 nucleotides in length that are involved in the regulation of a variety of biological or pathological processes through RNA interference (RNAi) [20]. A single miRNA can target multiple genes involved in cellular signaling pathways with potentially limited off-target effects, making then attractive targets for molecular cancer therapy [21]. Several miRNAs have been found to play pivotal roles in the chemotherapeutic resistance of PDAC [22,23,24,25,26]. Even further, several studies have reported that miRNAs can modulate the response to chemotherapy in pancreatic and other cancer types through their involvement in autophagy [27,28,29,30,31,32,33], evidencing the importance of post-transcriptional regulation by miRNAs in autophagy-induced drug resistance, and suggesting miRNA modulation as a potential therapeutic approach against the effects of cancer-induced autophagy.

Our group and others found that microRNA miR-198 acts as a tumor suppressor across multiple cancer types and is involved in the regulation of various oncogenic factors involved in migration, proliferation, induction of apoptosis, and drug resistance [34,35]. Overexpression or exogenous delivery of miR-198 leads to profound phenotypic effects including decreased cell proliferation, migration, and invasion in vitro and reduced tumor progression in xenograft tumors in vivo [36]. Among the validated targets of miR-198 is Valosin-containing protein (VCP) [36,37], also known as transitional endoplasmic reticulum ATPase (TER ATPase) and p97. VCP is a ubiquitously expressed protein belonging to the AAA+ family (ATPases associated with various activities). It is involved in multiple cellular processes, including cell cycle regulation, nuclear envelope formation, Golgi biogenesis, the ubiquitin proteasome system, and the degradation of misfolded substrates synthesized through the secretory pathway [38]. Increased VCP protein expression is observed in many cancers and correlates with poor patient outcomes and has been reported to play an important role in autophagy [39,40,41,42], implicating VCP as a promising therapeutic target for which several allosteric and ATP-competitive small molecule inhibitors have been developed with encouraging anti-tumor activity in pre-clinical models [43]. Given that VCP is directly targeted by miR-198, we sought to determine whether VCP repression through miR-198 replacement may potentially disrupt the autophagy process and sensitize PDAC cells to gemcitabine treatment.

Despite significant advances in the field, the effective and safe delivery of miRNA mimics and other nucleic acids in vivo has remained elusive, with dose-limiting toxicities and a lack of efficacious drug delivery limiting the broad application of RNAi-based therapies [44] We have developed a novel drug delivery system that combines polyethyleneimine (PEI) with polylactic-co-glycolic acid (pLGA) to generate LGA-modified PEI, a novel polymer that can condense with nucleic acids through electrostatic interaction to form self-assembled LGA-PEI nanoparticles (LPNPs). LPNPs are characterized by high nucleic acid loading, high transfection efficiency in varied cell types, and low cytotoxicity in vitro and in vivo [45,46].

In this study, we examine whether miR-198-mediated downregulation of VCP may contribute to autophagy disruption and identify a potential mechanism through which miR-198 administration can sensitize pancreatic cancer cells to gemcitabine treatment. We then examine the clinical implications of the therapy by testing both efficacy and safety in vivo and determine whether LPNP-mediated delivery of miR-198 can synergize with gemcitabine to effectively reduce tumor burden and metastatic progression with a favorable toxicity profile.

## 2. Materials and Methods

### 2.1. Cell Culture, Stable Cell Lines, Plasmids, Chemicals, and Antibodies

Human MIA-PaCa2 (CRL-1420) and AsPC1 (CRL-1682) PDAC cell lines were purchased from ATCC, Manassas, VA, USA, and authenticated by DNA fingerprinting at the University of Texas MD Anderson Cancer Center Characterized Cell Line Core, Houston, TX, USA. Chloroquine (CQ) was purchased from Sigma-Aldrich, St Louis, MO, USA. The MIA-MSLN cell line is a MIA-PaCa2 cell line with forced MSLN overexpression and was generated as previously described [47,48]. Subsequently, stable cells overexpressing miR-198 or vector control cells were generated from MIA-MSLN cells (MIA-MSLN-miR-198, MIA-MSLN-miR-Ctrl) and AsPC-1 cells (AsPC1-miR-198 and AsPC1-miR-Ctrl), with the Lenti-miR miRNA Precursor Clone Collection, System Biosciences, Palo Alto, CA, USA, as previously described [36]. Lenti-miR-198 (p198, MI0000240) plasmid and Lenti-miR-CTL (pCtrl, CD511B-1) were also purchased from System Biosciences, Palo Alto, CA, USA. VCP expression plasmid (pVCP) was purchased from OriGene, Rockville, MD, USA, and the mRFP-GFP-LC3 (pLC3) plasmid was furnished by Dr. Mary K. Estes at Baylor College of Medicine, Houston, TX, USA. Rabbit anti-MSLN polyclonal antibodies were custom-made by Genemed Inc., San Francisco, CA, USA and characterized as previously described [48]. Matrigel™ Membrane Matrix was purchased from Corning, Corning, NY, USA. MSLN immunohistochemistry (IHC) staining antibody MB-G10 was purchased from Rockland Immunochemicals, Limerick, PA, USA and anti-VCP antibody (Ab) from GeneTex, Irvine, CA, USA. Anti-LC3 Ab was ordered from Novus Biologicals, Littleton, CO, USA. Anti-Ki67 Ab goat anti-rabbit IgG (H&L) antibody-horseradish peroxidase conjugate and goat anti-mouse IgG (H&L) antibody-horseradish peroxidase conjugate were obtained from Santa Cruz Biotechnology, Dallas, TX, USA. Anti-α-Tubulin Ab was from Sigma-Aldrich, St Louis, MO, USA.

### 2.2. Preparation of LGA-PEI Polymer

LGA-PEI polymer was formulated as previously described [45,46]. Briefly, The LGA-PEI polymer was prepared by directly mixing PLGA (12–16 kDa) and branched PEI (B-PEI) (25 kDa) in organic solvent. Specifically, 250 mg B-PEI and 120 mg PLGA were dissolved separately in 10 mL tetrahydrofuran (THF) each, combined, and then moderately stirred at room temperature for 48 h. The soft precipitate was separated from the THF solution and washed with THF solvent two times. The solid was then dried in a vacuum at room temperature overnight.

### 2.3. Production and Characterization of Nucleic Acid-Containing Nanoparticles (LPNPs)

LPNPs were formulated as previously described [45,46]. Briefly, for in vitro studies, 10 μg of either pRFP, p198, pVCP, or pCtrl in 50 μL of ultrapure water was added to 25 μg of LGA-PEI polymer in 50 μL of ultrapure water and incubated at room temperature for 30 min before use. For in vivo studies, an approximate dose of 2.5 mg/kg for an average 20 g mouse was used; 50 μg of either p198 or pCtrl in 100 μL of ultrapure water was added to 125 μg of LGA-PEI polymer in 100 μL of ultrapure water and incubated at room temperature for 30 min before use.

The size and the zeta potential of LPNPs were determined using a Zetasizer Nano ZS90 at a controlled temperature of 25 °C (Malvern Panalytical). For size measurement, the operational parameters were wavelength = 658 nm, angle = 90°, reference index fluid = 1.33, viscosity = 0.89 cP, and scattered light detection ≤ 300 kcps. For zeta potential measurement, disposable folded capillary cells, Malvern Panalytical, Malvern, UK, and the Smoluchowski approximation (F(κa) = 1.5) were used. The operational parameters were reference index fluid = 1.33, viscosity = 0.89 cP, dielectric constant = 78.5, and the dispersant viscosity was used as sample viscosity. In addition, the size was also analyzed with SEM imaging. Briefly, 50 μL of the LPNP dispersion was dropped onto a carbon tape mounted on an aluminum mount. After drying in the air at room temperature, the samples were coated with gold-palladium at 15 mA for 40 s to minimize surface charging. SEM imaging was conducted with a Hitachi S-5500 SEM instrument, Hitachi Instruments Inc., San Jose, CA, USA, at an accelerating voltage of 10 kV.

### 2.4. In Vitro Transfection Assay

Cell lines (2.5 × 10^5^ per well) were seeded into 6-well plates 24 h before transfection. For each well, either LPNP-p198, LPNP-pVCP, or LPNP-pCtrl formulations containing 10 μg of pDNA were diluted in 300 μL Opti-MEM Invitrogen, Carlsbad, CA, USA and added to cells growing in Opti-MEM medium, replacing the media after 6 h.

### 2.5. Cell Viability Assay

After LPNP-p198 transfection, cell lines were plated onto 96-well plates at 5000 cells/well and cultured in normal media overnight. The cells were treated with different doses of gemcitabine from 0 to 100 μg/mL and, as a comparative control, CQ-treated cells were evaluated. The cells were then tested in the 3-(4,5-dimethylthiazole-2-yl)-2,5-biphenyl tetrazolium bromide (MTT) assay as previously described [47]. Briefly, 20 μL of MTT reagent mixed with 200 μL of growth medium was added to each well and incubated at 37 °C for 4 h. Absorbance was recorded at 590 nm with an EL-800 universal microplate reader, Bio-Tek Instruments, Winooski, VT, USA. Cytotoxicity was expressed as the percentage of surviving cells relative untreated controls.

### 2.6. Autophagy Characterization

ASPC1-miR-198/Ctrl and MIA-MSLN-miR-198/Ctrl cells were grown on coverslips transfected with pLC3 using the Lipofectamine™ 2000 reagent (Invitrogen), according to the manufacturer’s instructions. At 24 h post transfection, cells were treated with 20 μg/mL gemcitabine for another 24 h. AsPC1 and MIA-MSLN cells were also treated with 10 μM CQ. Cells were then evaluated under a fluorescence microscope, Olympus Corp, Tokyo, Japan. Images under each channel or combined channels were acquired with Slide book software v4.0, Intelligent Imaging Innovations, Denver, CO, USA.

Pearson’s correlation coefficient was used for colocalization analysis by measuring the overlap of the pixels as follows:

$$r_{p}=\frac{\sum \left[\left(R_{i}-R_{avg}\right)\left(G_{i}-G_{avg}\right)\right]}{\sqrt{\sum {\left(R_{i}-R_{avg}\right)}^{2}\sum {\left(G_{i}-G_{avg}\right)}^{2}}}$$

where $R_{avg}$ and $G_{avg}$ are the averages of the red and green channels, respectively, and the summations with index i are over all the image voxels. The value of $r_{p}$ is between −1 and 1 and describes how well the red and green channels are related by a linear equation G=mR+b. The closer $r_{p}$ is to +/− 1.0, the more linearly (directly or inversely) related are all the voxel intensities in the two channels.

Ten random high-power fields of each transfected cell line were selected to quantify the extent of colocalization of green and red signals.

### 2.7. Pancreatic Cancer Mouse Models

For the CDX models, 3 × 10^6^ MIA-MSLN cells were injected orthotopically into the tail of the pancreas of 5- to 6-week-old male nude mice Charles River, Wilmington. MA, USA, as previously described [49]. We allowed two weeks for tumor establishment and used GFP fluorescence in tumor cells as a rough indicator of tumor formation. Mice were randomized into four groups: no treatment (Saline solution), gemcitabine, and LPNP-p198 or LPNP-Ctrl with gemcitabine. The treatment regimen commenced after tumor establishment. Gemcitabine was administered intraperitoneally at a concentration of 50 mg/kg bodyweight LPNP treatments were administered intravenously 2 times/week for 3 weeks or until mice appeared moribund and had to be sacrificed. The week after treatment, mice were euthanized and evaluated macroscopically for the presence of tumors and/or metastases in the abdominal cavity. Tumor spread was further visualized for GFP with a fluorescence filter. Tumor nodules and other organ tissues were explanted, weighed, and stored in RNALater solution Ambion, Austin, TX, USA at −80 °C for subsequent analysis. For the PDX model, fresh human PDAC specimens were obtained from surgical resections in Elkins Pancreas Center, Baylor College of Medicine, Houston, TX, USA. Primary tumor tissues (PDX F0) were cut into approximately 1 mm^3^ fragments and one fragment was subcutaneously implanted into each SCID/Beige mouse. When primary outgrowths (PDX F1) reached 10 mm in diameter, the tumor was harvested, and new fragments were re-implanted into new hosts. The PDX line was defined as stable upon growth at transplant generation 3 (PDX F3). PDX F3 tumors having a size of 1 mm^3^ were implanted subcutaneously into the right flank of 5- to 6-week-old male SCID/Beige mice. We allowed 3–4 weeks for tumor establishment. Mice were randomized into two groups, each receiving LPNP-Ctrl or LPNP-p198 together with gemcitabine. Treatments were administered intratumorally 2 times/week for 3 weeks or until mice appeared moribund and had to be sacrificed. The week after treatment, mice were euthanized and evaluated macroscopically for the presence of tumor and/or metastases.

For both pancreatic cancer tumor models, tumor size was measured with digital calipers and tumor volume was determined with the following formula:

Tumor volume (mm^3^) = [length (mm)] × [width (mm)]^2^ × 0.52

### 2.8. In Vivo Subchronic Toxicity Evaluation

Five-week-old Crl: CD-1^®^ IGS female mice, Charles River, Wilmington, MA, USA, were housed under specific pathogen-free conditions at a AAALAC (American Association for Accreditation of Laboratory Animal Care)-certified animal facility. All procedures related to the maintenance of animals and experiments were in strict accordance with the policy of the Internal Committee for the Care and Use of Animals (IACUC). Mice were randomly distributed into three experimental groups (four mice per group): saline solution (untreated), LPNP-p198, and LPNP-pCtrl. Administration was performed intravenously via the tail vein at a final dosing volume of 200 μL based on maximum bolus delivery volume for mice and at a dosing regimen of three times per week for six weeks. A daily general examination was carried out to determine any adverse effects caused by the treatment and to determine, if necessary, a humanitarian endpoint. After completion of the treatment regimen, blood parameters were assessed using a veterinary blood chemistry analyzer VetTest^®^ 8008, IDEXX, Westbrook, ME, USA.

### 2.9. MiRNA and mRNA Extraction and Reverse-Transcription

Total miRNA was extracted from tissues/cells and purified with the mirVana miRNA Isolation kit Ambion, Austin, TX, USA, following the manufacturer’s instructions. Specifically, 5 μL of RNA was directly converted into cDNA with the QuantiMir™ RT System System Biosciences, Palo Alto, CA, USA. Total mRNA was extracted with the RNAqueous RNA Isolation kit Ambion, Austin, TX, USA. Two micrograms of RNA were converted to cDNA with the iScript cDNA synthesis kit purchased from Bio-Rad, Hercules, CA, USA. The QuantiMir System from System Biosciences, Palo Alto, CA, USA was used to measure miR-198 expression, normalized to U6. Briefly, cDNAs from different cell lines and tissue samples were mixed with SYBR^®^ Green Mastermix Bio-Rad, Hercules, CA, USA. plus the universal reverse primers. Expression levels of mature miRNA were evaluated with a comparative threshold cycle (Ct) method and normalized to that of U6 (2^−ΔΔCt^).

### 2.10. Western Blot Analysis

PDAC tumor tissues were homogenized in RIPA buffer Sigma-Aldrich, St. Louis, MO, USA, with a protease inhibitor cocktail purchased from Boehringer Mannheim, Basel, Switzerland. Total proteins (20 μg) of each sample were loaded into 10% or 15% (for LC3 detection only) SDS-polyacrylamide gradient gels. Proteins were then transferred onto nitrocellulose membranes Bio-Rad, Hercules, CA, USA and detected with specific primary antibodies, and appropriate HRP-conjugated secondary antibodies in the ECL detection system Amersham Biosciences, Amersham, UK.

### 2.11. Hematoxylin-Eosin and Immunohistochemistry Staining

Tumor or other tissues were routinely processed, embedded in paraffin, and stained with hematoxylin-eosin. Histological features, including the growth pattern of tumor cells, cell morphology, and mitotic features, were evaluated. Immunohistochemistry was performed on paraffin-embedded sections from all mouse tumor tissues. We used the standard ABC method with a panel of antibodies: Ki67 Santa Cruz Biotechnology, Dallas, TX, USA, MSLN (MB-G10, diluted 1:50), Rockland Immunochemicals, Limerick, PA, USA,, and VCP (Polyclonal, diluted 1:200), GeneTex, Irvine, CA, USA. Corresponding positive and negative control samples were used. The Ki67 labeling index was determined as the percentage of immunoreactive tumor cells in the evaluated area. Scoring of VCP and MSLN was graded as three scales based on the intensity and percentage of cells with cytoplasmic staining: score 0—negative cytoplasmic staining for all tumor cells; score 1—weak cytoplasmic staining in all tumor cells other than score 2; score 2—moderate cytoplasmic staining >50% or strong cytoplasmic staining in >5% of tumor cells [50].

### 2.12. TUNEL Assay

Apoptosis was evaluated by measuring DNA fragmentation with the DeadEnd™ Colorimetric TUNEL System, Promega Corporation, Madison, WI, USA, for TUNEL (TdT-mediated dUTP Nick-End Labeling). Briefly, paraffin-embedded tissue sections were deparaffinized in fresh xylene and rehydrated by immersion in graded ethanol. Sections were then treated with proteinase K and washed in PBS. Fixation was repeated in 4% paraformaldehyde in PBS. After equilibration with equilibration buffer, 100 µL of TdT reaction mix was added to tissue sections on slides and incubated at 37 °C for 60 min. Detection of reactive nucleotides was performed with Streptavidin HRP DAB substrate reaction. The positive control was a sample treated with DNAase, and the negative control a sample incubated without rTdT enzyme.

### 2.13. Statistical Analysis

All experiments were conducted at least in triplicate. Statistical analyses were carried out using the SPSS Statistics v23.0 statistical package, IBM, Armonk, NY, USA. Normality and variance homogeneity parameters were estimated using the Shapiro–Wilk test and the Levene test, respectively. The significant differences between the groups were estimated using the Student t-test or ANOVA in the case of parametric data, and the Mann–Whitney or Kruskal–Wallis U test in the case of non-parametric data. A value of *p* > 0.05 was considered statistically significant.

## 3. Results

### 3.1. LGA-PEI Nanoparticles (LPNPs) Efficiently Deliver Functional miR-198 into Pancreatic Cancer Cells In Vitro

Characterization of LPNPs demonstrated suitable physicochemical properties for efficient delivery of nucleic acids, including an average zeta potential of 65.1 mV and average diameters 113.5 nm when measured using Scanning Electron Microscopy, and of 229.4 nm with a polydispersity index (PDI) of 0.115 when measured using Dynamic Light Scattering (Supplementary Figure S1).

Our previous study showed that reconstitution of miR-198 can downregulate the expression of MSLN [36]. To demonstrate functionally relevant LPNP-mediated delivery in vitro, we used MIA-PaCa2 cells stably overexpressing MSLN. Transfection with red fluorescent protein plasmid (pRFP) using LPNPs demonstrated cellular uptake and functional release of nucleic acids (Supplementary Figure S2a). Transfection with miR-198 plasmids (LPNP-p198) resulted in marked upregulation of miR-198 expression compared to controls as measured by qRT-PCR (Supplementary Figure S2b), as well as a significant reduction in MSLN mRNA and protein levels (Supplementary Figure S2c,d). These data demonstrate that LPNP-p198 can effectively deliver functional miR-198 into PDAC cells.

### 3.2. In Vitro Administration of miR-198 Sensitizes PDAC Cells to Gemcitabine Treatment through VCP Downregulation-Mediated Autophagy Maturation Process Inhibition

Our group and others have previously shown that VCP is a direct target of miR-198 [36,37]. It has also been reported by several groups that VCP plays an important role in autophagy formation, and disruption can inhibit autophagosome maturation to autolysosome [42]. In addition, accumulated evidence has shown that autophagy is activated in PDAC cells and that it is one of the mechanisms responsible for the refractory response of pancreatic tumors to gemcitabine treatment [51]. We hypothesized that miR-198 could inhibit autophagy through downregulation of VCP, leading to sensitization of PDAC cells to gemcitabine treatment.

**Figure 1:**
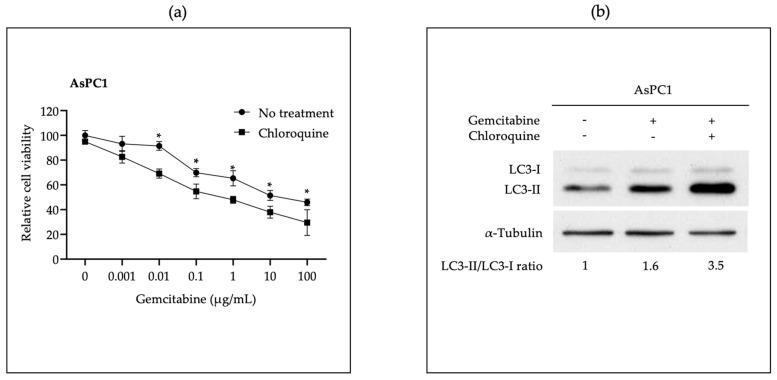

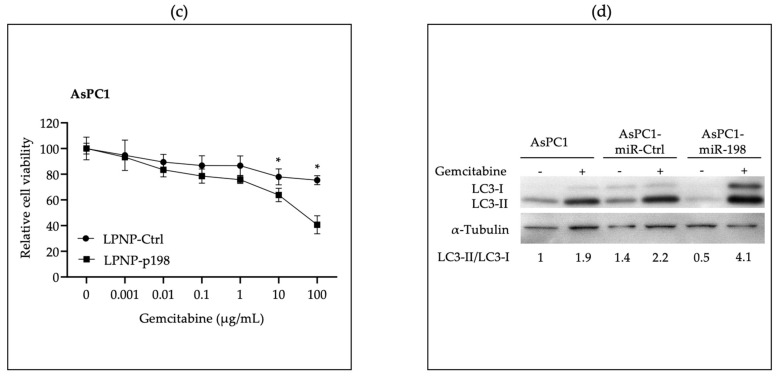
In vitro restitution of miR-198 sensitizes AsPC-1 cells to gemcitabine treatment through the inhibition of the autophagy process. (**a**) Treatment with autophagy inhibitor chloroquine (CQ) sensitizes AsPC-1 cells to gemcitabine toxicity. AsPC-1 cells were treated with different concentrations of gemcitabine in the presence or absence of CQ (10 µM). Cell viability was compared with that of untreated control, which is set at 100% (n = 5, * *p* < 0.05). (**b**) Increased accumulation of LC3-II in CQ-treated AsPC-1 cells upon gemcitabine treatment. AsPC-1 cells were treated with gemcitabine for 24 h with or without the autophagy inhibitor CQ (10 µM), and autophagy markers LC3-I and LC3-II were determined with Western blot. Both LC3-II and LC3-I bands were normalized to α-tubulin and the normalized LC3-II/LC3-I ratios indicated. (**c**) MiR-198 reconstitution sensitizes AsPC-1 cells to gemcitabine treatment. AsPC-1 cells were transfected with LPNP-p198 or LPNP-Ctrl for 24 h, and then treated with different concentrations of gemcitabine (0, 0.001, 0.01, 0.1, 1, 10, and 100 μg/mL) for 48 h. Cell survival was then determined with the MTT assay. Cell viability was compared with that of untreated control, which is set at 100% (n = 5, * *p* < 0.05). (**d**) MiR-198-overexpressed AsPC-1 cells show an autophagy inhibition effect like that of CQ treatment. Three cell lines, AsPC-1, AsPC-1-miR-CTRL, and AsPC-1-miR-198, were treated with gemcitabine for 24 h and autophagy markers LC3-I and LC3-II were determined.

We first confirmed inhibition of the autophagy maturation process with hydroxychloroquine (CQ), an autophagy inhibitor that disrupts autophagosome maturation by diffusing into lysosomes and impairs them through protonation-wise alkalinization of the lumen [52], increasing the sensitivity of PDAC cells to gemcitabine treatment. We demonstrated that addition of CQ sensitized AsPC-1 cells to gemcitabine cytotoxicity in a dose-dependent manner (Figure 1a), and that the ratio of LC3-II/LC3-I increased in gemcitabine plus CQ-treated AsPC-1 cells, when compared with the gemcitabine-only group (Figure 1b). These results indicate that an autophagy alteration is happening and, therefore, sensitizing PDAC cells to gemcitabine treatment.

Next, we tested whether the effect of miR-198 on PDAC cells was like that of CQ in the autophagy maturation process. We transfected LPNP-p198 or LPNP-Ctrl into AsPC-1 cells and subsequently treated them with increasing concentrations of gemcitabine. The growth of cells treated with LPNP-p198 was significantly inhibited by gemcitabine treatment when compared with LPNP-Ctrl treatment (Figure 1c). We also used stable cell lines overexpressing miR-198 or vector control (AsPC-1-miR-198 and AsPC-1-miR-CTL) together with parental cell line AsPC-1 to evaluate the LC3-II/LC3-I ratio in the presence or absence of gemcitabine. All cell lines showed an increase in LC3-II expression after gemcitabine treatment; however, AsPC-1-miR-198 had an even higher LC3-II/LC3-I ratio (Figure 1d). These results indicate that miR-198 may function like CQ, affecting the autophagy process.

**Figure 2:**
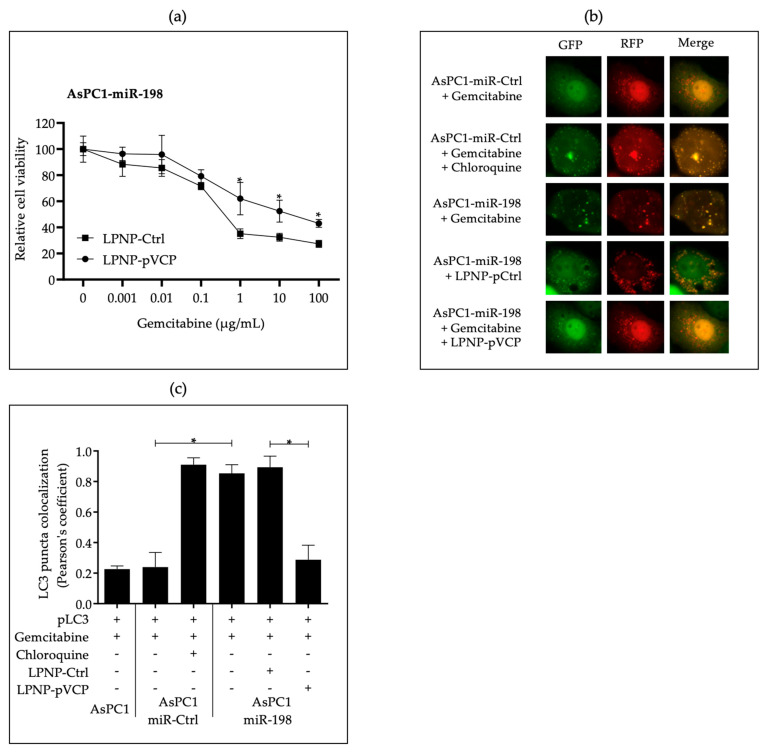
In vitro administration of miR-198 impairs the autophagy maturation process and reverses when VCP is overexpressed. (**a**) AsPC-1-miR-198 cells were transfected with either LPNP-Ctrl or LPNP-pVCP and then treated with different concentrations of gemcitabine (0, 0.001, 0.01, 0.1, 1, 10, and 100 μg/mL) for 48 hrs. Cell viability was determined with the MTT assay (n = 5, * *p* < 0.05). AsPC-1-miR-CTL and AsPC-1-miR-198 were transfected with LPNP-Ctrl or LPNP-pVCP together with mRFP-GFP-LC3 and cultured in complete medium with gemcitabine (20 µg/mL). Cells were also cultured in the presence or absence of CQ (10 µM). (**b**,**c**) LC3 expression was then visualized with fluorescence microscopy under green channel, red channel, and both channels overlapped. These are representative pictures taken from at least three different replica experiments and, for quantification of LC3-RFP-GFP expression via red and green puncta colocalization, Pearson’s correlation coefficient was used. Ten independent fields of cells with more than thirty cells in each field were counted for each panel of cells (n = 5, * *p* < 0.05).

VCP is involved in multiple activities throughout the autophagic pathway, including autophagy activation through the regulation of several transcription factors; autophagy initiation through promotion of formation and stabilization complexes; and autophagy maturation through cooperation in autophagosome and lysosome fusion [42]. We determined whether sensitization of PDAC cells to gemcitabine treatment resulted from miR-198-mediated repression of VCP. Using the MTT assay, we found that cells with forced VCP overexpression regained resistance to gemcitabine toxicity when compared with vector control cells, suggesting that autophagy was taking place in VCP-rescued cells (Figure 2a). Next, we monitored the autophagy maturation process in cells forced to express miR-198 using an mRFP-GFP-LC3 indicator plasmid to determine whether autophagosomes were fusing with lysosomes to become autolysosomes following constitutive miR-198 expression. Because the low pH inside the autolysosome quenches GFP, only RFP LC3 puncta can be detected when indicative of a complete autophagy maturation process. However, if the process of autophagosome to autolysosome conversion is blocked, expressed LC3 becomes trapped in the autophagosome under neutral conditions, and therefore, both RFP and GFP-LC3 proteins can be observed as yellow puncta, indicating an abnormal autophagy. When cells were treated with CQ, we observed more yellow puncta, indicating an interrupted autophagy maturation process. Similarly, in miR-198-overexpressing cells we observed more yellow puncta with very few red puncta, indicating that autophagy maturation was similarly disrupted in miR-198-overexpressed cells after gemcitabine treatment. In the cells transfected with exogenous VCP, more red puncta appeared after gemcitabine treatment as compared with the vector control cells, indicating a normal autophagy process and chemoresistance to gemcitabine (Figure 2b,c). Our results demonstrate that miR-198 can inhibit the autophagosome maturation process via VCP downregulation, thereby sensitizing PDAC cells to gemcitabine treatment.

Mesothelin (MSLN) is a glycosylphosphatidylinositol (GPI)-anchored cell surface protein which is overexpressed in ~85% of human pancreatic cancer cells and clinical specimens [53]. Overexpression of MSLN results in increased proliferation, migration, and invasion in vitro and increased tumor growth and progression in vivo, as well as in a marked reduction in endogenous miR-198 levels [36,54]. We repeated the above experiments in MIA-MSLN cells to replicate and corroborate the above findings in the context of another pancreatic cancer cell line (Supplementary Figure S1).

### 3.3. In Vivo Delivery of miR-198 Sensitizes PDAC Cells to Gemcitabine through Downregulation of VCP-Mediated Autophagosome Maturation, Leading to a Significant Reduction in Tumor Burden and Metastases

**Figure 3:**
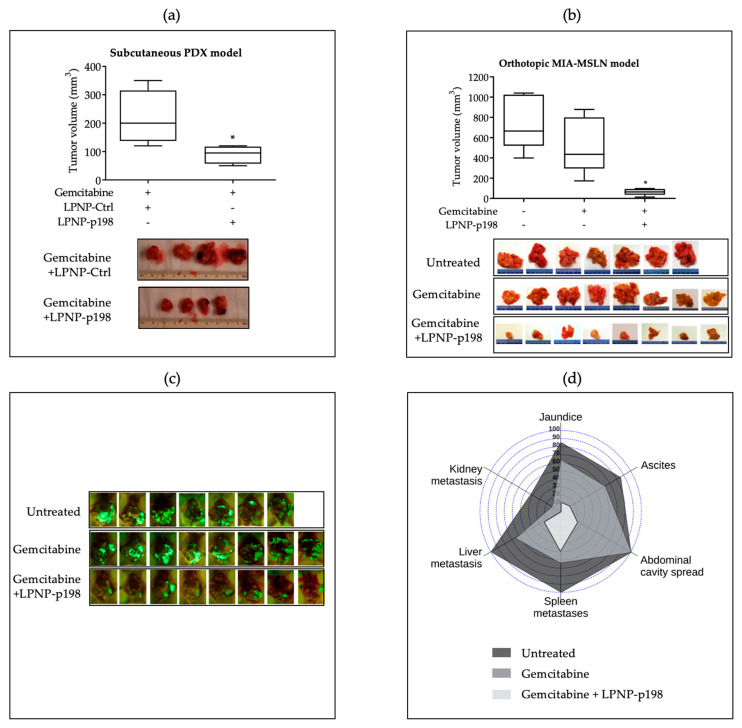
In vivo delivery of miR-198 sensitizes PDAC cells to gemcitabine, leading to a significant reduction in tumor burden and metastases. (**a**) SCID/Beige mice received a subcutaneous implant of a stable transplant from a patient-derived tumor to establish a xenograft, and then received an intratumoral injection of either LPNP-p198 or LPNP-Ctrl in combination with intraperitoneal injection of gemcitabine (50 mg/kg) 3x/wk for 2 weeks. Both tumor weight and volume indicated a significant tumor regression in the LPNP-p198 group compared with those of LPNP-Ctrl group (n = 6 and n = 4, respectively. * *p* < 0.05). (**b**–**d**) Nude mice received an orthotopic implant of MIA-MSLN cells and two weeks later began to receive intraperitoneal injection of gemcitabine (50 mg/kg) (once per wk for 3 weeks) in combination with a tail vein injection of LPNP-p198 (3x/wk for 3 weeks). The animals were sacrificed after the treatment and tumor size was analyzed with fluorescence imaging and gross dissection. (**b**) Tumor volume (n = 8 and n = 7, respectively. * *p* < 0.05), (**c**) fluorescence imaging of GFP^+^ tumor cells, and (**d**) clinical signs and metastatic nodule count indicated a significant tumor regression and reduction in comorbidities and metastases in the LPNP-p198 group compared with those of the control group.

With the support of in vitro data indicating that miR-198 can sensitize PDAC cells to gemcitabine treatment, we tested the potential for therapeutic delivery of miR-198 in combination with gemcitabine using a polymeric LPNP delivery system for nucleic acids. We first tested whether LPNPs condensed with miR-198 plasmids (LPNP-p198) could effectively enter tumor tissue and improve therapeutic outcome in vivo through direct tumor injection. Studies attempting to test the therapeutic potential of autophagy inhibitors in pancreatic cancer have found a highly variable response that appears to be highly differentiated across cell lines [55]. We therefore wanted to recapitulate our results in the context of the heterogenic nature of PDAC using a patient-derived xenograft (PDX) model from a tumor resistant to gemcitabine. Using direct tumor injection of LPNP-p198, we were able to confirm a marked reduction in tumor weight when given in a combination regimen with miR-198 compared to gemcitabine alone (Figure 3a). 

Having shown that direct injection of LPNP-p198 inhibited tumor growth in subcutaneous PDAC mouse models, we next sought to investigate the clinically relevant question of whether systemic, intravenous delivery of LPNP-p198 could reduce tumor size and metastatic spread of PDAC cells in an established orthotopic PDAC tumor model. We previously reported that miR-198 administration in pancreatic cancer cells leads to a significant reduction in tumor burden and metastases [36]. We now examined whether miR-198 therapy could exert a clinically relevant effect in the context of gemcitabine resistance when administered post tumor establishment through LPNPs. We found that tail-vein injection of LPNP-p198 sensitized orthotopic pancreatic tumors to gemcitabine treatment and significantly reduced tumor burden and metastatic spread when compared with controls (Figure 3b,c). Treatment with LPNP-p198 plus gemcitabine not only resulted in a significant and pronounced reduction in tumor weight, but this group also had the fewest mice with jaundice, ascites, and metastases of the abdominal cavity, spleen, liver, and kidney (Figure 3d), indicating that this combination has promising therapeutic potential from multiple perspectives.

**Figure 4:**
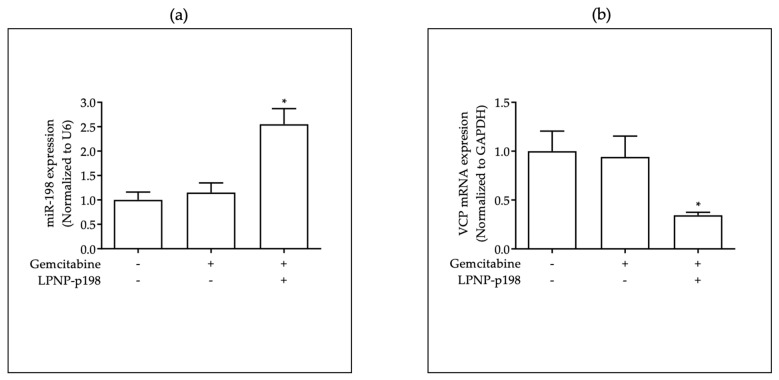

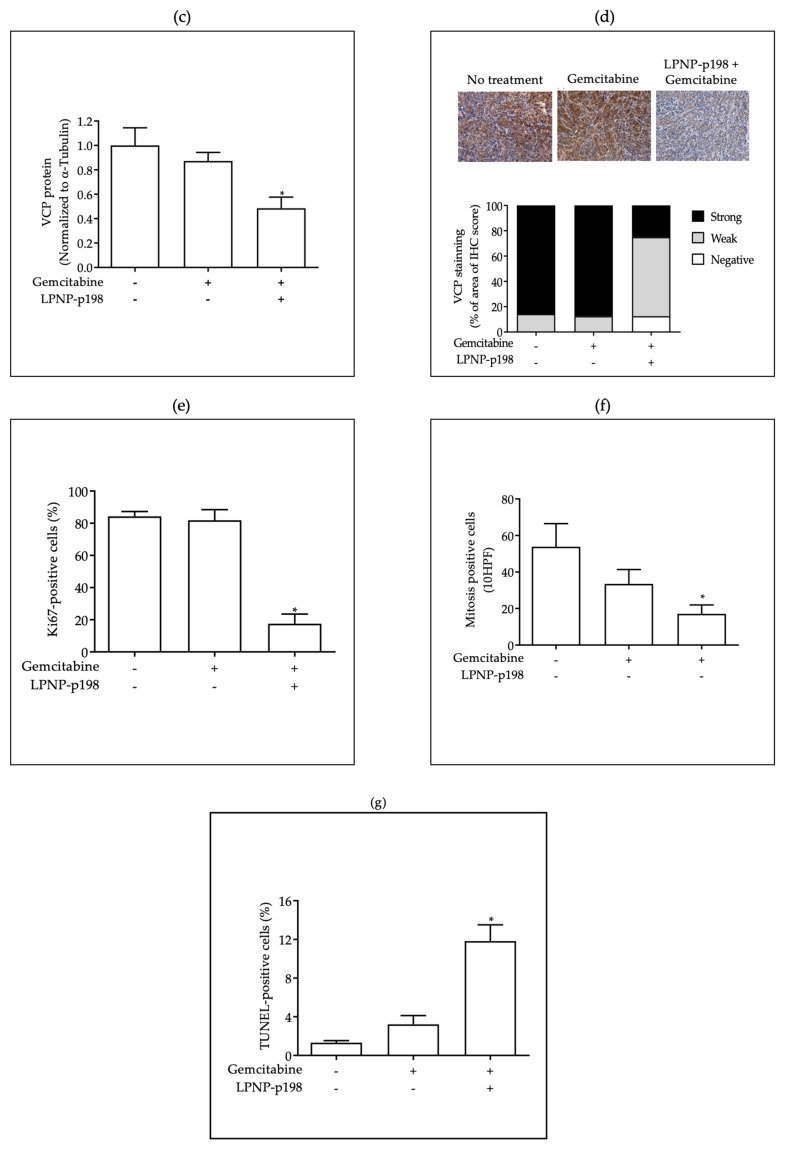
In vivo downregulation of VCP expression through restitution of miR-198 at tumor cells leads to a significant reduction in tumor aggressiveness capabilities when combined with gemcitabine. (**a**) miR-198 expression, (**b**) VCP mRNA expression, (**c**) VCP protein expression, and (**d**) IHC staining of VCP protein levels of the orthotopic xenograft tumor cells indicated restitution of miR-198 and downregulation of VCP in the LPNP-p198 group compared with those of control group. Concomitantly, (**e**) IHC staining of Ki67, (**f**) number of mitosis positive cells, and (**g**) TUNEL staining of apoptotic cells indicated significant reduction in tumor aggressiveness capabilities in the LPNP-p198 group compared with those of control group (n = 8 and n = 7, respectively. * *p* < 0.05).

To support our hypothesis that miR-198 precipitates PDAC cell sensitization to gemcitabine treatment through downregulation of VCP, we determined the levels of miR-198 and VCP expression in the treated and untreated xenograft tumor tissue and found that when miR-198 levels were increased through LPNP-mediated delivery (Figure 4a), both VCP mRNA (Figure 4b) and VCP protein (Figure 4c) levels were significantly downregulated and inversely correlated with miR-198 expression in mice treated with LPNP-p198 plus gemcitabine. Moreover, quantitative IHC staining positive scores (Figure 4d) indicated that VCP expression levels were significantly reduced in the group treated with LPNP-p198 plus gemcitabine.

Then, to further characterize the anti-tumor effects of combination therapy in vivo, we studied the effects of LPNP-p198 therapy on cell proliferation and apoptosis in the xenograft tumor tissue. We observed a significant reduction in Ki67-positive cells and mitosis numbers in tumor tissues from mice treated with LPNP-p198 plus gemcitabine, indicating that the proliferation rate of tumor cells was markedly reduced as compared with controls (Figure 4e,f). In addition, a TUNEL assay revealed a significant increase in the number of apoptotic cells in tumor tissues from mice treated with LPNP-p198 plus gemcitabine compared to controls (Figure 4g). These data suggest that targeting VCP through miR-198 modulation could be a new therapeutic strategy affecting drug resistance in pancreatic cancer.

### 3.4. LPNPs Demonstrate a Favorable Safety Profile When Administered Systemically in a Repeated Dose Toxicity Study

Having shown that intravenous injection of LPNP-p198 inhibited tumor growth in an orthotopic PDAC mouse model through VCP-downregulation-mediated sensitization to gemcitabine treatment, we sought to further delve into the clinical relevance of LPNP-mediated therapy by conducting a safety profile of liver- and kidney-function-related parameters following LPNP-p198 treatment at the efficacious dosing regimen tested. We conducted a subchronic study to evaluate the effect of miR-198-loaded LPNP administration on the blood parameters of CD-1 mice following three intravenous administrations per week for six weeks. No significant changes were observed in the blood parameters for liver and kidney function in any of the animals, as shown in Table 1. Likewise, we observed no changes in body weight measurements in the treated mice as compared to the controls (Supplementary Figure S4).

## 4. Discussion

At the time of diagnosis, most PDAC patients are already in the later stages of the disease and have missed the opportunity for surgery. Gemcitabine is a first-line treatment option for patients with locally advanced or metastasized unresectable tumors and provides important clinical benefits to patients, especially when combined with Nab-paclitaxel [19,56]. However, most tumors are either inherently resistant to gemcitabine or become so, which is one of the main reasons why PDAC patients have poor outcomes. Increasing evidence has reflected that dysregulation of miRNA expression is closely associated with chemoresistance in PDAC. Several recent pre-clinical studies have identified select miRNAs, such as members of the miR-200 family and miR-21, that contribute to gemcitabine resistance [57,58,59]—with some playing multiple roles through targeting of different factors. For example, miR-33a contributes to enhancing gemcitabine sensitivity by downregulating Pim-3 kinase [23], while also suppressing the translocation of β-catenin [24]. Certain microRNAs directly contribute to resistance through their involvement in autophagy [26,27,29,32] and may be overexpressed or repressed by processes including lncRNA-mediated sponging [28,30,33,60].

Accumulating evidence implicates that having an active autophagy process may contribute to tumor progression. Autophagy switches its role from tumor suppressor to tumor promoter once malignant tumors emerge, with cancer cells adapting to exploit autophagy for survival during stressful conditions such as nutrient deprivation, hypoxia, or cytotoxic insults triggered by cancer therapy [51,61,62]. Gemcitabine resistance is related to increased autophagy, and gemcitabine itself is known to induce autophagy, which in turn increases cell survival and induces drug resistance [63], leading to the promising use of inhibitors of autophagy as a therapeutic modality. Autophagy is a dynamic process comprised of three sequential steps: formation of autophagosomes, fusion of autophagosomes with lysosomes, and degradation of entrapped cargo [64]. Prior studies demonstrated that VCP is a key player in the autophagy process from phagosome initiation and elongation to autolysosome maturation [42], and loss of VCP activity impairs autophagy, due to reduction in the PtdIns3P production [65,66], destabilization, and fragmentation of lysosomes [67] and accumulation of dysfunctional autophagosomes with increased size [68]. The targeting of VCP activity has therefore emerged as a promising therapeutic option in cancer. Several allosteric and ATP-competitive small molecule inhibitors have been developed in recent years [69]. MiR-198 is an exonic miRNA transcribed in the 3′UTR of Follistatin-like 1 (*FSTL1*), which plays a role in wound healing [70]. It has also attracted a lot of attention as an integral tumor suppressive player in several cancers, where it targets several key factors involved in the complex tumorigenic regulatory network, and exogenous administration of miR-198 in vitro and in vivo leads to a reduction in proliferation, migration, and invasion in multiple cancer types; for a review, see [34,35]. Since VCP is a direct target of miR-198 [36,37], we examined whether exogenous administration of miR-198 could alter autophagy through VCP targeting and sensitize cancer cells to gemcitabine.

In line with previous findings, we observed that autophagy inhibitor CQ sensitized cells to gemcitabine (Figure 1a and Supplementary Figure S1) and increased the LC3-II expression, and thus LC3-II/LC3-I ratio (Figure 1b). We also further determined a similar sensitization to gemcitabine (Figure 1c) and a LC3-I, LC3-II, and LC3-II/LC3-I ratio increase (Figure 1d) in cells with forced miR-198 expression. While in a different context, these results have been reported previously by Yu et al. using a hepatocyte-derived carcinoma cell line transfected with a miR-198-inducible expression system [71]. Since autophagy is a dynamic process, increase in either LC3-I, LC3-II, or the LC3-II/LC3-I ratio cannot be specifically associated with either autophagic flux, autophagosome formation, or autophagic degradation; instead, it should be supported with other confirmatory analysis [72].

**Figure 5:**
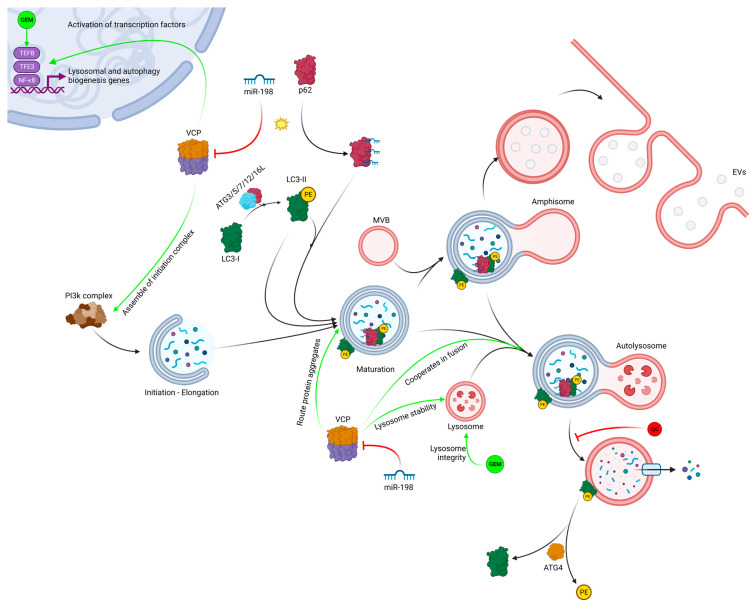
Overview of different VCP functions potentially regulated by miR-198 in a gemcitabine-mediated autophagy scenario, based on our observations and current literature [42,71,73]. In the phagosome formation steps, miR-198 may regulate (I) autophagy biogenesis-related genes through downregulation of VCP-mediated activation of transcription factors, (II) phagosome initiation and elongation through VCP-mediated PI3K complex formation and stabilization, and (III) protein aggregate degradation through VCP-mediated protein aggregate routing to the phagosome. In the autolysosome maturation steps, miR-198 may regulate proper phagosome content degradation through VCP-mediated lysosomal stability and phagosome–lysosome fusion. This diagram also contains additional information on mechanisms by which endogenous miR-198 may be exported in EVs through p62/LC3-II-mediated miR-198 incorporation into multivesicular bodies. Created with BioRender.com.

To further support the hypothesis that miR-198 can sensitize PDAC cells to gemcitabine due to the disruption of autophagy through VCP-mediated targeting, we performed a rescue experiment by introducing exogenous VCP without a miR-198 targeting site in its 3′UTR and found they regained resistance to gemcitabine toxicity when compared with vector control cells, suggesting that autophagy was taking place in VCP-rescued cells (Figure 2a). Given that VCP is a key player in multiple parts of autophagic flux, these results suggest that miR-198-mediated VCP modulation may play a role at various points in the autophagy process, both through LC3-I accumulation due to impaired phagosome formation and IC3-II accumulation due to impaired autolysosome formation. A graphical representation of the process can be found in Figure 5.

To further explain the observed increase in the LC3-II/LC3-I ratio, we performed a mRFP-GFP-LC3 puncta formation assay, which shows that both RFP and GFP were in the same puncta in miR-198-overexpressing cells, suggesting that autophagy maturation was inhibited in these cells in a similar way to that observed in cells treated with CQ, while RFP fluorescence was the one that predominated in control cells, including the VCP-rescued cells (Figure 2b,c and Figure 5).

In this study, we delivered miR-198 in vivo both intratumorally and systemically using our novel LPNP-based platform, with a focus potential clinical translation of miR-198-mediated modulation of autophagosome maturation. The combination of intratumoral miR-198 administration and gemcitabine in resistant cells resulted in improved outcomes with regards to tumor burden compared to gemcitabine alone in a heterogenically representative subcutaneous PDX model (Figure 3a). To approach clinical application more closely, we tested the viability of using LPNPs for systemic intravenous administration of miR-198 in the context of enhancement of gemcitabine sensitivity. Treatment with LPNP-p198 plus gemcitabine not only resulted in a significant and pronounced reduction in tumor burden (Figure 3b,c), but this group also had the fewest mice with jaundice, ascites, and metastases of the abdominal cavity, spleen, liver, and kidney (Figure 3d). In support of the potential clinical application of nanoparticle-mediated miR-198 therapy, we examined the preliminary safety profile of intravenous administration of LPNPs in a subchronic exposure experiment with a dosing regimen of three times per week for six weeks. No significant changes were recorded in either the daily general examinations or blood parameters, demonstrating a favorable safety profile in a dosing regimen that was extended to be three times longer than the efficacious dosing regimen tested (Table 1). Further studies to establish a minimum effective dose and maximum tolerated dose are currently underway.

To further support our hypothesis that miR-198 precipitates PDAC cell sensitization to gemcitabine treatment through downregulation of VCP, we determined the levels of miR-198 and VCP expression in treated and untreated tumor tissues and found that when miR-198 levels were increased through LPNP-mediated delivery (Figure 4a), both VCP mRNA (Figure 4b) and VCP protein (Figure 4c) levels were significantly downregulated and inversely correlated with miR-198 expression in mice treated with LPNP-p198 plus gemcitabine. Moreover, quantitative IHC staining positive scores (Figure 4d) indicated that VCP expression levels were significantly reduced in the group treated with LPNP-p198 plus gemcitabine. Then, we further characterized the anti-tumor effects of combination therapy in vivo by studying the effects of LPNP-p198 therapy on cell proliferation and apoptosis in the xenograft tumor tissue. We observed a significant reduction in Ki67-positive cells (Figure 4e) and cells undergoing mitosis (Figure 4f), and a concomitant increase in apoptotic cells (Figure 4g) in tumor tissues from mice treated with gemcitabine plus LPNP-p198. A therapeutic regimen using LPNP-p198 followed by gemcitabine thereby effectively inhibits tumor cell proliferation and induces tumor cell apoptosis in an orthotopic PDAC mouse model through miR-198-mediated VCP regulation.

Our study presents a novel therapeutic strategy for targeting autophagy-mediated resistance in pancreatic cancer and lays the groundwork for its application in other resistant cancer types, given that prior studies have also determined that targeting VCP can also sensitize cells to cisplatin and other treatments [74]. Autophagy is constitutively activated in oncogenic KRAS-driven tumors and is thought to be necessary for their development [75]. While recent data support autophagy inhibition as an approach to sensitizing PDAC to gemcitabine [55], the results both in vitro and in vivo appear to be highly variable and cell-type dependent. The autophagy inhibitor verteporfin, for instance, only moderately enhanced the antitumor activity of gemcitabine in a pancreatic ductal adenocarcinoma model [18], and while recent clinical studies have had positive results, others have fared only moderately [76]. There are currently no sensitive and reliable predictive biomarkers to identify patients who could most likely benefit from autophagy inhibition. The use of miRNA-based approaches might provide additional advantages in this regard, given their inherent promiscuity as regulators. A single miRNA such as miR-198 may target multiple genes involved in different cellular signaling pathways. This makes miRNAs such as miR-198, miR-29a [29], and miR-33 [23,24] key targets for the development of potential multi-pronged treatments that can regulate a complex tumorigenic network through a central vantage point. Using autophagy-targeting miRNAs such as miR-198 will likely still have an impact on tumorigenesis, even in tumors that are not dependent on autophagy, and would be expected to have an even more significant impact on tumors through their effects exerted on other targets.

## 5. Conclusions

We have shown therapeutic delivery of miR-198 using LGA-PEI as a delivery system resulted in repression of VCP-associated autophagy and sensitized PDAC to gemcitabine treatment, reducing tumor burden and metastatic spread in PDAC mouse models. Furthermore, a favorable preliminary safety profile was established following subchronic exposure extending beyond the efficacious dosing regimen. This work establishes the use of LGA-PEI as a prototype for safe and effective nucleic acid delivery in vitro and in vivo, with potential to be used from the bench to the clinic.

## Figures and Tables

**Table 1:** Effects of LPNPs on blood parameters of CD-1 mice after three intravenous administrations per week for six weeks.

Parameter (Units)	Group (Expressed in Mean ± S.D., n = 4)		
	Untreated (Saline Solution)	LPNP-pCtrl (2.5 mg/kg)	LPNP-p198 (2.5 mg/kg)
Albumin (g/dL)	2.4 ± 0.1	2.2 ± 0.2	2.4 ± 0.2
Globulin (g/dL)	2.6 ± 0.3	2.5 ± 0.1	2.6 ± 0.2
Albumin/Globulin (ratio)	0.94 ± 0.07	0.88 ± 0.12	0.91 ± 0.04
Total protein (g/dL)	5.0 ± 0.5	4.6 ± 0.1	5.1 ± 0.2
Alkaline phosphatase (U/L)	42 ± 7	49 ± 10	39 ± 8
Alanine transaminase (U/L)	56 ± 21	53 ± 8	45 ± 5
Lipase (U/L)	848 ± 103	845 ± 81	826 ± 48
Amylase (U/L)	1565 ± 164	1557 ± 77	1500 ± 83
Creatine (mg/dL)	0.08 ± 0.1	0.10 ± 0.08	0.13 ± 0.1
Urea nitrogen (mg/dL)	0.20 ± 0.14	0.18 ± 0.1	0.23 ± 0.15
Total bilirubin (mg/dL)	21 ± 2	20 ± 1	19 ± 2
Glucose (mg/dL)	171.3 ± 29.3	156.8 ± 7.7	157.0 ± 17.8

## Data Availability

All data collection was conducted in accordance with ethical guidelines. Data and protocols are available upon request.

## Supplementary Materials

The following supporting information can be downloaded at: https://www.mdpi.com/article/10.3390/pharmaceutics15082038/s1, Figure S1: Physicochemical properties of LPNPs. (a) Dynamic Light Scattering analysis of LPNPs size shows an average diameter of 229.4 nm and an average PDI of 0.115. (b) Zeta potential analysis of LPNPs shows an average charge of 65.1 mV. (c) Scanning electron microscopy imaging of LPNPs shows an average diameter of 113.5 nm; Figure S2: LPNPs efficiently deliver miR-198 to PDAC cells in vitro. (a) LPNPs demonstrate efficient delivery of RFP expressing plasmids in MIA-MSLN cells, as visualized using fluorescence; (b) qRT-PCR assay detects a significant increase in miR-198 levels in MIA-MSLN cells transfected with LPNP-p198 than controls (LPNP-pCtrl). (c) qRT-PCR and (d) western blot analyses demonstrate a significant reduction in MSLN mRNA and protein levels, respectively, following LPNP-p198 treatment (n = 3, * *p* < 0.05); Figure S3: In vitro restitution of miR-198 sensitizes the MSLN high expressing PDAC cell line MIA-MSLN to gemcitabine treatment through VCP downregulation-mediated autophagy maturation process inhibition. (a) MIA-MSLN cells were transfected with LPNP-p198 or LPNP-Ctrl for 24 h, and then treated with different concentrations of gemcitabine (0, 0.001, 0.01, 0.1, 1, 10 and 100 μg/mL) for 48 h. Cell survival was then determined with the MTT assay. Cell viability was compared with that of untreated control, which is set at 100% (n = 5, ** p* < 0.05). (b) MIA-MSLN -miR-198 cells were transfected with either LPNP-Ctrl or LPNP-pVCP. Cells were then treated with different concentrations of gemcitabine (0, 0.001, 0.01, 0.1, 1, 10 and 100 μg/mL) for 48 h. Cell viability was determined with the MTT assay (n = 5, * *p* < 0.05). (c,d) MIA-MSLN -miR-CTL, MIA-MSLN-miR-198 were transfected with LPNP-Ctrl or LPNP-pVCP together with mRFP-GFP-LC3 and cultured in complete medium with gemcitabine (20 µg/mL). Cells were also cultured in the presence or absence of CQ (10 µM). (c) LC3 expression was then visualized with fluorescence microscopy under green channel, red channel, and both channels overlapped. These are representative pictures taken from at least three different replica experiments. (d) For quantification of LC3-RFP-GFP expression via red and green puncta colocalization, Pearson’s correlation coefficient was used. Ten independent fields of cells with more than 30 cells in each field were counted for each panel of cells (n = 5, * *p* < 0.05); Figure S4. Effects of LPNPs on body weight of CD-1 mice after three intravenous admin-istrations per week for six weeks.
