# Supplementary material for: Nanoparticle-Mediated Therapy with miR-198 Sensitizes Pancreatic Cancer to Gemcitabine Treatment through Downregulation of VCP-Mediated Autophagy
Source: Pharmaceutics. 2023 Jul 28;15(8):2038. doi: 10.3390/pharmaceutics15082038 (PMC10457905; doi:10.3390/pharmaceutics15082038)
Supplement: Supplementary file 1 [file pharmaceutics-15-02038-s001.zip › pharmaceutics-2472061-supplementary.pdf]

Supplementary Materials

(a)

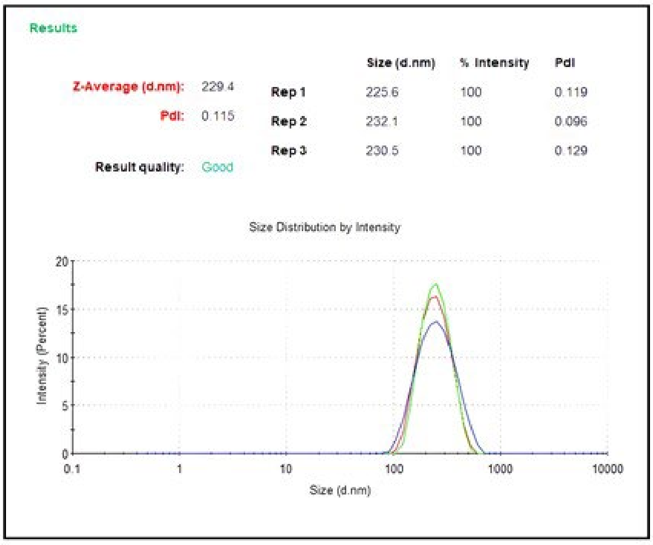

(b)

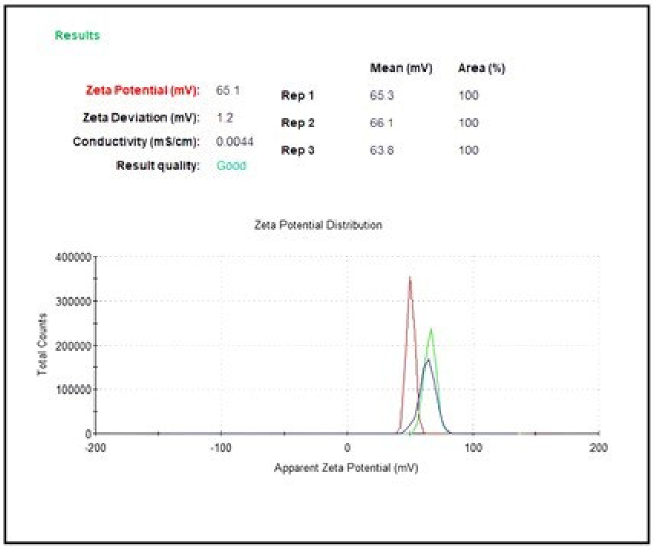

(c)

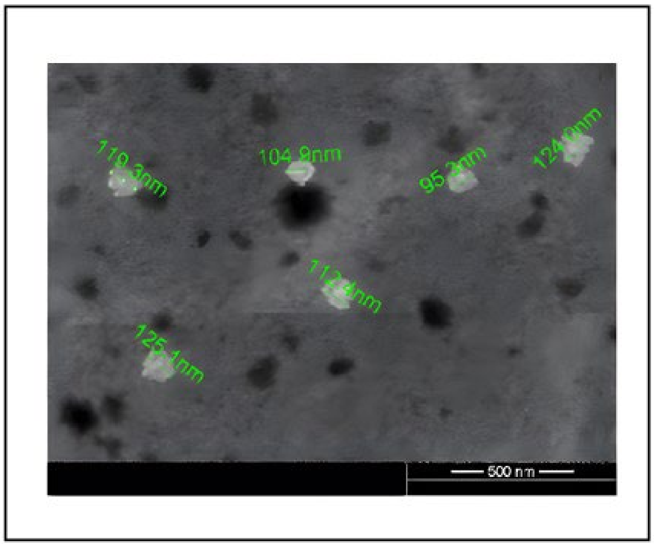

Figure S1. Physicochemical properties of LPNPs. (a) Dynamic Light Scattering analysis of LPNPs size shows an average diameter of 229.4 nm and an average PDI of 0.115. (b) Zeta potential analysis of LPNPs shows an average charge of 65.1 mV. (c) Scanning electron microscopy imaging of LPNPs shows an average diameter of 113.5 nm.

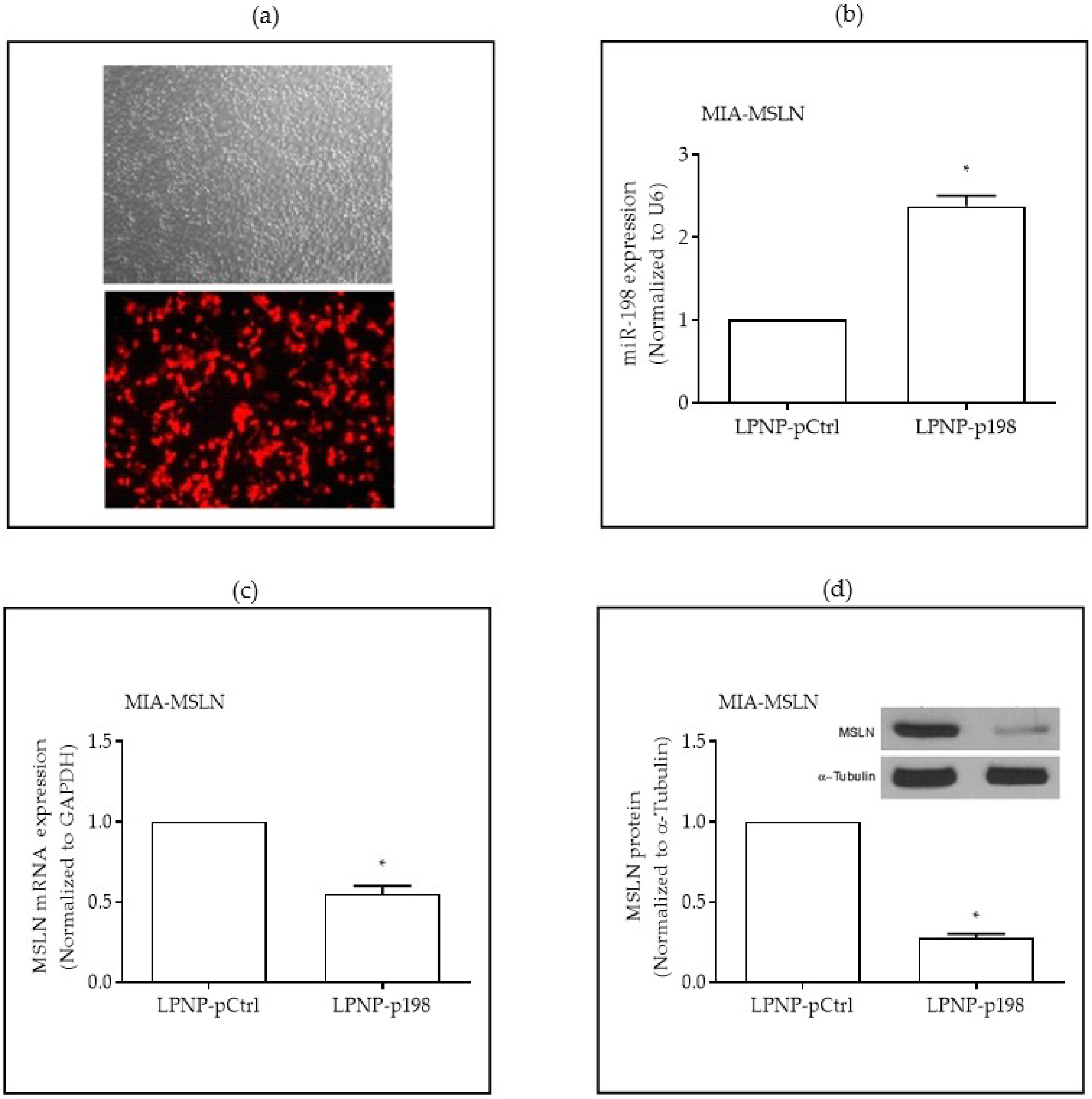

Figure S2. LPNPs efficiently deliver miR-198 to PDAC cells in vitro. (a) LPNPs demonstrate efficient delivery of RFP expressing plasmids in MIA-MSLN cells, as visualized using fluorescence; (b) qRT-PCR assay detects a significant increase in miR-198 levels in MIA-MSLN cells transfected with LPNP-p198 than controls (LPNP-pCtrl). (c) qRT-PCR and (d) western blot analyses demonstrate a significant reduction in MSLN mRNA and protein levels, respectively, following LPNP-p198 treatment (n = 3, * $p < 0.05$).

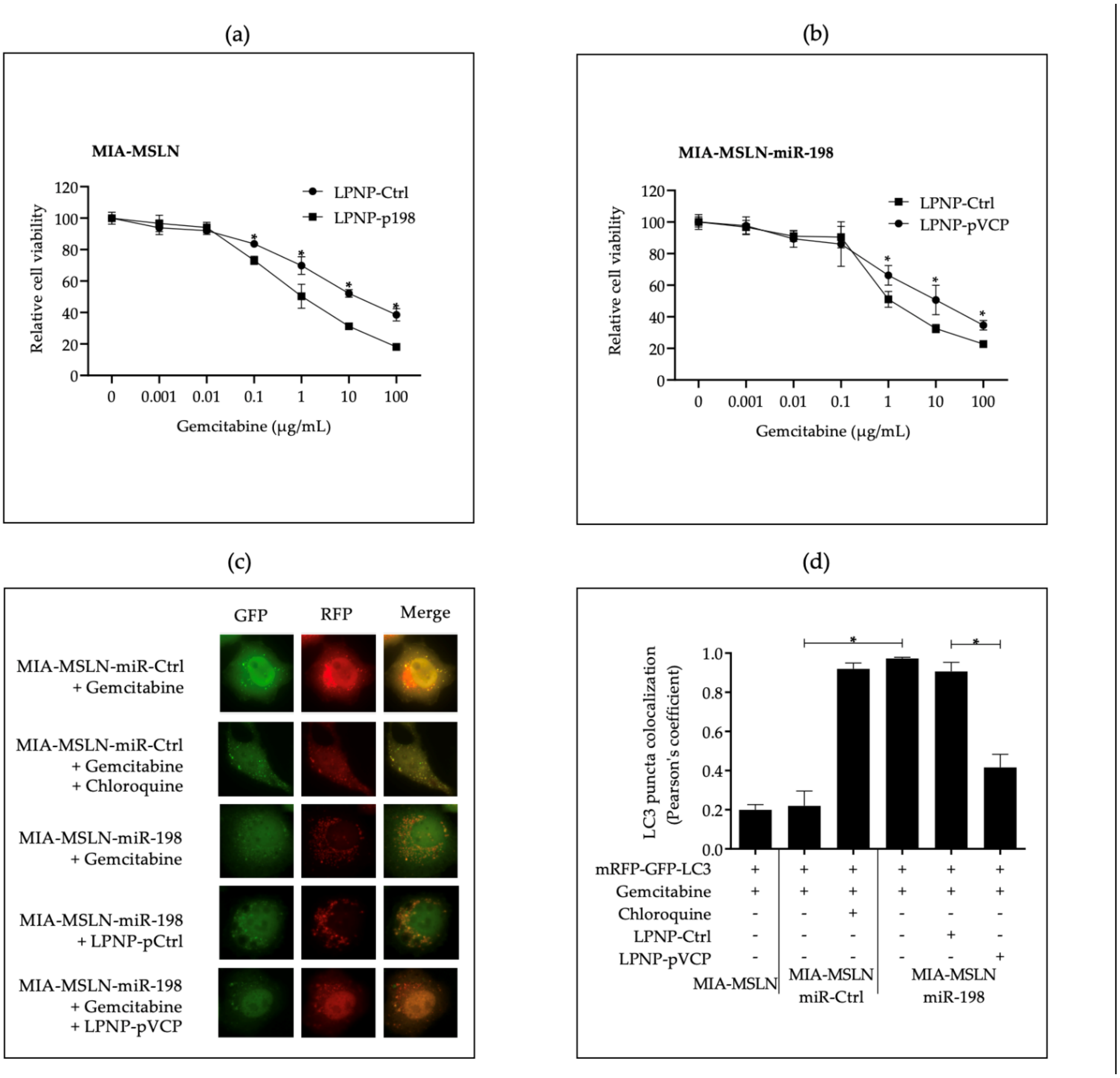

Figure S3. In vitro restitution of miR-198 sensitizes the MSLN high expressing PDAC cell line MIA-MSLN to gemcitabine treatment through VCP downregulation-mediated autophagy maturation process inhibition. (a) MIA-MSLN cells were transfected with LPNP-p198 or LPNP-Ctrl for 24 h, and then treated with different concentrations of gemcitabine (0, 0.001, 0.01, 0.1, 1, 10 and 100 μg/mL) for 48 h. Cell survival was then determined with the MTT assay. Cell viability was compared with that of untreated control, which is set at 100% (n = 5, * $p < 0.05$). (b) MIA-MSLN -miR-198 cells were transfected with either LPNP-Ctrl or LPNP-pVCP. Cells were then treated with different concentrations of gemcitabine (0, 0.001, 0.01, 0.1, 1, 10 and 100 μg/mL) for 48 h. Cell viability was determined with the MTT assay (n = 5, * $p < 0.05$). (c, d) MIA-MSLN -miR-CTL, MIA-MSLN-miR-198 were transfected with LPNP-Ctrl or LPNP-pVCP together with mRFP-GFP-LC3 and cultured in complete medium with gemcitabine (20 μg/mL). Cells were also cultured in the presence or absence of CQ (10 μM). (c) LC3 expression was then visualized with fluorescence microscopy under green channel, red channel, and both channels overlapped. These are representative pictures taken from at least three different replica experiments. (d) For quantification of LC3-RFP-GFP expression via red and green puncta colocalization, Pearson's correlation coefficient was used. Ten independent fields of cells with more than 30 cells in each field were counted for each panel of cells (n = 5, * $p < 0.05$).

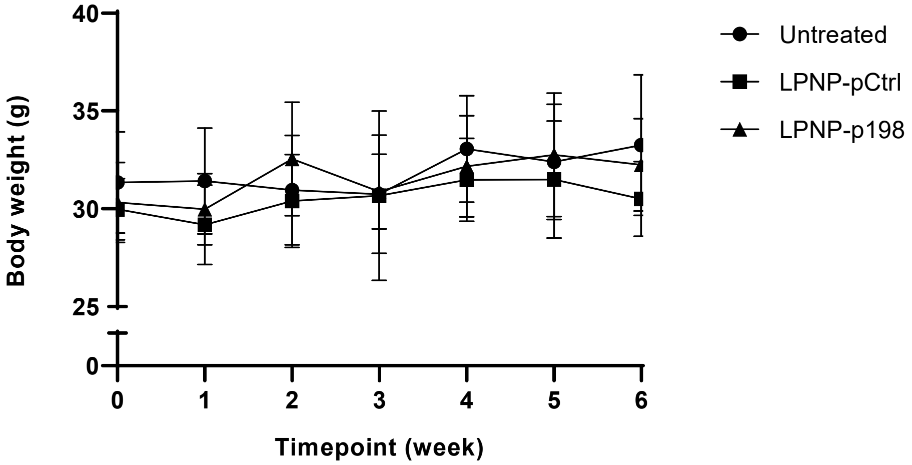

Figure S4. Effects of LPNPs on body weight of CD-1 mice after three intravenous administrations per week for six weeks.
